# Out of area placements for people with “Personality Disorder”: Making the case for a local intensive psychotherapeutic alternative

**DOI:** 10.1002/pmh.1649

**Published:** 2024-11-25

**Authors:** Susan Mizen, Vanessa Jones, Susan Howson

**Affiliations:** ^1^ Devon Partnership NHS Trust The University of Exeter School of Psychology; ^2^ School of Psychology and Counselling The Open University Milton Keynes UK; ^3^ Partnership NHS Trust

## Abstract

**Objectives:**

The use of Out of Area (OoA) psychiatric placements for people with “Personality Disorder” (PD) is widespread in the UK. An innovative local intensive psychotherapeutic service, adapted to the transdiagnostic presentations of the most complex PD patients, likely to be placed out of the area, was devised in the English County of Devon. This paper reports the findings of a Freedom of Information (FOI) request to commissioners attempting to quantify PD OoA placements in England and the cost offset of the local therapeutic alternative to OoA placements in Devon.

**Design and Methods:**

Data from FOI requests was combined with publicly available sources to assess the scale of OoA placements for people with PD in England. OoA service use and cost data were used to examine the cost offset and effectiveness of the local alternative to OoA placements in Devon.

The results found a lack of transparency and excessive use of OoA placements despite UK Government intentions. Data from the local therapeutic service demonstrated cost‐effectiveness, reducing OoA placements and increasing the availability of psychotherapeutic services.

This paper suggests the number of OoA placements can be reduced for people with severe and complex PD. The local therapeutic service provides a model for future rehabilitation pathways.

## INTRODUCTION

“Personality disorders”^1^(PD) are common mental health problems reported to comprise 33–52% of psychiatric outpatients and up to 50% of mental health inpatient services (Evans et al., 2017). A small minority with the most severe and complex “Borderline PD” (BPD), present with persistent para‐suicidal behavior, co‐morbid eating disorders, and medically unexplained physical symptoms (Blasco‐Fontecilla et al., 2009; Macina et al., 2021; Sansone et al., 2005). In the absence of community services tailored to their needs, these people are often detained as inpatients for lengthy periods for their own safety (Zanarini, 2001). One reason for this is that people with severe and complex BPD have commonly experienced multiple adverse childhood experiences (Zanarini, 2000) requiring intensive psychotherapeutic help or therapeutically informed support (NICE Guideline 2009).

However, their risk to self, disordered eating, and somatic symptoms often escalate in psychotherapy. PD specialists and community mental health services are not designed or funded to contain and manage the ensuing disturbance. Consequently, many of the most complex patients cannot access therapy, increasing their hopelessness and suicidality. Ultimately, high‐risk behavior is managed in acute mental health inpatient settings in which access to psychotherapy is also limited. When admissions become extended because the risks of discharge cannot be managed, a solution can be sought through referral to Out of Area (OoA) placement. These are not acute mental health placements but individually commissioned planned placements for patients assessed to require psychotherapeutic help in an inpatient setting. These services may be called Specialist Complex Needs services, PD inpatient services, or High Dependency Inpatient Rehabilitation services offering PD‐specific psychotherapies such as Dialectical Behavior Therapy (DBT) (Linehan, 2014). While NHS England funds some inpatient specialist PD services, demand far exceeds supply, so the majority are provided by the private sector and funded on a case‐by‐case basis by local Integrated Care Boards (previously Clinical Commissioning Groups or CCGs) using NHS and social care funding.

For the sake of clarity, it is important to distinguish elective long‐term OoA placements for people with PD from the use of acute OoA beds when the number of NHS mental health beds is insufficient to meet demand. This has been an increasingly common practice as the number of NHS beds has fallen. A recent report by the Royal College of Psychiatrists found the number of mental health beds in England fell by 73% from 67,100 in 1987/88 to less than 18,400 currently (Strategy Unit, Midlands and Lancashire Commissioning Support Unit, 2020). The use of acute Mental Health OoA beds is associated with substantially worse outcomes for length of stay, self‐harm, and number of contacts with services following discharge than local mental health beds (Galante et al., 2019) and so is considered unacceptable and poor value for money (Mahase, 2019; Royal College of Psychiatrists, 2019). The Government stated ambition was “to eliminate inappropriate^2^ OoA placements for adults in acute mental health inpatient care by 2020–21” (Department of Health & Social Care, 2016; NHS Digital, 2023). Despite this ambition, the number of acute OoA placements continues to rise.

The excessive use of long‐stay OoA placements is also an area of concern (Ryan et al., 2004). In 2016/17 the Care Quality Commission (CQC) expressed concern about the high number of beds in OoA rehabilitation placements highlighting excessive use of detention under the Mental Health Act and the absence of effective local co‐ordinated rehabilitation pathways back to community settings, resulting in people receiving excessively restrictive services many miles away from home (CQC, 2018). However, this report did not enquire about diagnosis and the subsequent development of rehabilitation pathways focused on the needs of patients with psychotic disorders. The absence of routine data collection leads to a lack of transparency about differences between areas, length of stay, cost, and treatment. So, the true extent of OoA placements for people diagnosed with PD is unknown and there is no national strategy toward developing local rehabilitation pathways. Other obstacles to the development of pathways for people with severe and complex PD include an absence of clinically effective therapeutic models for the severe PD group with transdiagnostic presentations in community settings. Finally, the high costs of care and perceived costs of implementing alternative pathways result in inaction by both commissioners and providers. There is little evidence in the academic literature to inform decision‐making about people with severe and complex PD in inpatient settings.

Regarding the cost of illness, high service use and social costs have been found among people with PD compared with other mental health problems (Bender et al., 2001; Cailhol et al., 2016; Penner‐Goeke et al., 2015; Soeteman et al., 2008; van Asselt et al., 2007), the highest costs relating to hospital treatment (Wagner et al., 2022), no studies to date have focused on the costs of hospitalization for the severe and complex PD group. Evidence for the cost‐effectiveness of therapeutic intervention for PD inpatients is scarce. Recent studies of the clinical and cost‐effectiveness of the mentalization‐based treatment (MBT) (Blankers et al., 2023) comparing a day program with outpatient therapy were not targeted toward inpatients. Earlier studies of therapeutic community provided in inpatient settings with people with PD treated informally confirmed greater cost‐effectiveness when inpatient psychotherapy was followed by a step‐down pathway when compared with routine psychiatric care (Beecham et al., 2006; Chiesa et al., 2002). Dolan et al. (1996) also identified cost savings following informal inpatient treatment at the Henderson Hospital (Dolan et al., 1996). Just one small study specifically focused on a service for PD patients in OoA placements. This preliminary before and after evaluation of a PD case management service in a specialist community team for patients in OoA placements offering 100 weeks of Structured Clinical Management (Bateman & Fonagy, 2009) found a 100% reduction in OoA placements (n = 7), 80% reduction in admissions from a mean of 170.8 to a mean of 34.2 bed days being significant at the p < .05 level. With 42% increase in crisis team out‐of‐hours contacts from 8.4 to 12 in the first 12 months of the intervention, with an estimated cost saving of £2.2–2.6 M (Graham et al., 2019). The evidence of cost‐effectiveness of therapeutic services for the severe and complex hospitalized PD group is sparse and the need for further research and longer follow‐up studies have been highlighted (Brettschneider et al., 2014; Meuldijk et al., 2017; van Asselt et al., 2008; Wetzelaer et al., 2016; Wetzelaer et al., 2017; Wunsch et al., 2014).

This paper presents the results of two studies that aim to address these gaps in the literature. First, the findings of a study funded by the British and Irish Group for the study of Personality Disorder (BIGSPD: Zimbron et al., 2022) compiling data from CCGs about PD OoA placements. This is followed by a study of the cost‐effectiveness of a local intensive psychotherapeutic  specialist service commissioned to work with people in and ‘at risk’ of OoA placement. This service used a new psychotherapeutic model, the Relational Affective Model designed specifically for severe PD patients with transdiagnostic presentations (Mizen, 2014).

## AIMS

This paper addresses the following research questions:

1. Is it possible to obtain data on OoA placements for people diagnosed with PD in England?
  If so:
  1.1 To what extent are OoA placements used by PD patients?
  1.2 What costs and length of stay are associated with such placements compared with placements for patients diagnosed with psychosis?
  1.3 Who provides these placements and what treatment services are people discharged to?
2. Can a local alternative to OoA placements work effectively with people with PD who reach the NHS England‐defined threshold for inpatient treatment?
  2.1 Are the costs of this alternative therapeutic pathway offset by reductions in the number of OoA placements and length of stay?

## METHODS

### Research question 1: is it possible to obtain data on OoA placement for people diagnosed with PD in England?

#### Design

Data on OoA placements was collected by requests to local commissioners called Clinical Commissioning Groups (CCGs) under the Freedom of Information Act. The data request covered both acute OoA placements used when local mental health beds were not available and some, but not all, elective therapeutic and rehabilitation placements. The questions chosen were constructed by a research steering group comprising clinical practitioners and people with lived experience. CCGs were identified through the list published by NHS Digital (2020) for OoA placements. All CCGs in England at that time (191) were contacted for information for the period between 1st January 2017 and 31st October 2019, this being the time period in which CCGs were judged to have ready access to the relevant data.

They were asked the following questions:

1. Between the 1st of January 2017 to 31st of October 2019 how many OoA Mental Health placements did you make?
2. For each placement, please provide anonymized details as to:
  - How long the placement was initially contracted for?
  - How many days the placement actually last (or length of stay until 31st October 2019 if ongoing)?
  - How much the placement cost (costs to 31st October 2019 if ongoing)?
  - What was the primary diagnosis of the service user who was placed?
  - What were the other diagnoses of the service user who was placed?
  - Which organization provided the placement?
  - Was the status of the service user informal or detained under the Mental Health Act?
  - On discharge from placement, which local service was the service user referred to (if any)?

Analysis was planned to distinguish “Personality Disorder” placements from other mental health placements and to enable relevant comparisons of the responses to the above questions.

#### Data collection

The problems obtaining data are relevant to the first research question. There was wide variation in the responses to the FOI request and many delays in receiving data. Data collection was complicated by the reorganization of many CCGS while the study was in progress, e.g. clustering CCGs into one organization or geographical reorganization and lack of clarity about which organization held the data required. In a third of the cases, CCGs managed the data. In others, data was held by NHS Trusts. Some CCGs subscribed to Commissioning Support Units some of which held the data themselves while others referred the question back to the CCG. Responses were also delayed due to the Covid‐19 pandemic. Follow‐up requests and new requests were stopped due to time constraints once responses received represented 74% of the total number of bed days published by NHS Digital (2020).

Sixty‐two CCGs provided some level of data some of which had been amalgamated giving 40 data sets. Duplicate data was removed. Fifteen CCGs refused to give any information: 11 of these refused under Section 17(1) of the Freedom of Information Act, on the basis that the data would take more than 18 hours to provide but still refused when simpler data was requested. While several of these had a high number of Out of Area placement days (highest 7,535 days, lowest 290 days, average 2,450 days) other CCGs with higher numbers did manage to report data. Four CCGs were refused under Section 40(2) of the Freedom of Information Act, stating disclosure could lead to patient identification. In some but not all instances the number of placements was small so there was a strong rationale for refusal. In summary, the systems for recording information vary greatly between CCGs.

## RESULTS

### Question 1 is it possible to obtain data on OoA placements for people diagnosed with PD in England?

Data from the 62 CCGs who responded was analyzed, representing 53% of the total days/costs compared to data published by NHS England (NHS Digital, 2020). For the 34‐month period (1 January 2017 to 31 October 2019) CCGs reported in total of 7,328 placements with a total cost of £138,350,710 and an average bed night cost of £327.

#### Question 1.1 to what extent are OoA placements used by PD patients?

Only 22 CCGs reported diagnostic information accounting for just 2,732 of the total 7,238 placements. Therefore, we obtained diagnostic information on 37% of reported placements. ‘Psychotic and Delusional disorders’ accounted for 46% of placements, followed by ‘Personality disorders’ (14%): a breakdown of diagnoses is shown in Table 1.

**TABLE 1:** Number of out of area placements by diagnosis.

Diagnosis stated	No. of placements	% of total
Psychotic and Delusional disorders	1,266	46%
Personality disorders	383	14%
Depressive episode/disorder	195	7%
Bipolar disorder	184	7%
“In crisis”	139	5%
Dementia	99	4%
Self‐harm	92	3%
ASD/LD	84	3%
Physical	76	3%
Anxiety disorders	71	3%
Drug and alcohol difficulties	70	2%
Perinatal	23	1%
Other (including PTSD, eating disorders, CAMHS)	50	2%
Total	2,732	100%

Of the 53% of patients for whom diagnostic information was available 88% had been detained under the Mental Health Act (1983). Of those people diagnosed with a PD 67% were detained under the MHA, the remainder being in hospital informally.

### Question 1.2: what costs and length of stay are associated with such placements compared with placements for patients diagnosed with psychosis?

Since Psychosis and Personality Disorder were most frequently placed OoA we compared responses. Mean costs and length of stay were similar: the analysis is shown in Table 2. While the mean costs of placements were similar for patients with PD and psychosis there was a wider distribution of costs for patients with PD. The mean length of stay was also higher.

**TABLE 2:** Cost and length of stay by diagnosis.

Diagnosis	Cost (£)				Length of stay (days)			
	Mean	Min	Max	SD	Mean	Min	Max	SD
PD	29,169	70	741,000	60,741	74	1	670	107
Psychosis	30,286	‐	384,000	43,043	62	1	833	100

#### Question 1.3: who provides these placements and what treatment services are people discharged to?

Non‐NHS organizations provided 99% of OoA placements for people with PD, two providers (Priory Group and Cygnet Group) accounting for 71% of placements. Little information was provided about which services people were discharged to. In the majority of cases, people were discharged to Community Mental Health Teams except for four people who were offered no further service. None of those discharged were referred to specialist PD services.

## RESEARCH QUESTION 2

### Introduction

#### The Devon personality disorder specialist service (PDSS): a local psychotherapeutic alternative to OoA placement for people with PD.

The Devon PDSS offered the Relational Affective Model, an intensive psychotherapeutic approach. This was designed to meet the needs of people with the most severe PD who had been hospitalized because of their high risk of suicide and transdiagnostic presentations which had failed to respond to symptom‐specific care pathways (Mizen, 2014). The PDSS is described here both as a therapeutic innovation and to provide a context for the research findings. A business case was made identifying the cost of hospitalizing people with PD in mental and physical health services which led to the commissioning of a psychodynamic therapeutic pathway to address the needs of people at risk of OoA placement at a cost of £1.4 M p/a. The aim of the service was to improve therapeutic outcomes and redistribute resources from a small number of high‐cost patients to provide an integrated therapeutic pathway across mental health, physical health, and social care. The PDSS opened in 2011 and, following a phased implementation became fully operational in 2015.

A severity threshold was set to ensure the service only addressed the needs of the people with PD at risk of OoA placement. The threshold chosen was that required to access NHS England inpatient PD services, namely Health of the Nation Outcome Scales (Honos) Cluster 8 and Thames Valley Severity Score 8 (NHSE, 2014). The service worked with people already placed OoA preventing delayed discharges and supporting repatriation to local services. It also worked with people with PD in local inpatient or community settings for whom referral OoA was being considered with the aim of providing a less restrictive alternative closer to home. Between 2011 and 2020 the PDSS worked with 254 people: 119 appeared on the Individual Patient Placement Budget (IPP) which either funded their OoA inpatient services or supported accommodation in the county alongside the PDSS as an alternative to OoA admission. The remaining 135 people on the PD placement were diverted from placement and so did not appear on the IPP budget, receiving a therapeutic service without requiring inpatient or residential placement.

#### PDSS service design

The PDSS offered assessment, outreach, and a three‐year intensive therapeutic pathway. Assessment provided a ‘gatekeeping’ function finding local alternatives to OoA placement where possible. Where there was no alternative to OoA placement, the duration of placement and therapeutic task were defined along with a step‐down therapeutic pathway following discharge which was identified at the point of commissioning. The outreach team worked with people in OoA placements planning their return to the PDSS. It engaged people with PD in the county who were eligible for the service but not ready to start therapy. The outreach team supported accommodation providers to provide Psychologically Informed Environments (Haigh et al., 2012) and consulted with mental health and social care teams to optimize support and containment. The three‐year therapeutic pathway consisted of a one‐year, four‐day‐a‐week therapeutic community day program for those with the most severe difficulties followed by two years of outpatient psychotherapy. The adapted therapeutic community approach used across all three years was the Relational Affective Model which is described in full elsewhere (Mizen, 2014). In the day program patients were offered twice weekly individual psychodynamic therapy, group analytic psychotherapy, psychosocial nursing practice, and family therapy. In the outpatient program, they were offered once‐weekly individual and group psychodynamic therapy and a fortnightly psychosocial group alongside a lower level of housing support.

The service aimed to offer relational containment in place of the concrete containment provided by hospital admission. The term ‘relational containment’ refers to the proposed role of unconscious conflict in driving relational disturbance in the psychodynamic therapeutic model (Mizen, 2014). This disturbance relating to self and others is expressed as anxiety about survival and contained in the therapeutic community, requiring long‐term therapeutic relationships. Risk was assessed and managed in psychodynamic terms to contain the anxiety of the team and facilitate positive risk‐taking. As patients increasingly felt their difficulties were understood and there was a realistic chance of recovery, they became less suicidal.

## METHODS

### Research question 2

2.1 Can a local alternative to OoA placements work effectively with people with PD who reach the NHS England threshold for inpatient treatment?
2.2 Are the costs of this alternative therapeutic pathway offset by reductions in the number of OoA placements and length of stay?

### Design

Between 2011 and 2020 Devon Partnership NHS Trust (DPT) provided mental health services for a population of just under one million people. The Individual Patient Placement (IPP) team oversaw elective OoA placements, maintaining a database of funded placements. This database included all locally commissioned (CCG funded) mental health placements, both inpatient OoA placements and the health component of local high support housing offered as an alternative to or step down from OoA placement. The data included primary diagnosis, admission and discharge dates, and the cost of all elements of care including transport and nursing observations for CCG‐funded placements. More limited information was available for nationally commissioned placements (NHS England funded) for which admission and discharge dates were available but not financial information. This IPP database was used to identify the number of inpatient OoA and local housing placements for people with PD per year along with the costs of placement and length of stay for each year. The cost of providing the PDSS itself was taken directly from PDSS budget data.

### Data collection

Data was collected from the IPP database in the financial years between April 2011 when the PDSS first opened and April 2020 by searching for all non‐forensic placements with a primary diagnosis of PD and those with eating disorders where BPD was the secondary diagnosis. The search identified 131 people fulfilling these criteria. 119 (91%) were on the caseload of the PDSS. Thirteen patients (9%) were not either because they chose to live outside Devon on leaving specialist placement or because they were not referred. The number of people in placement, length of stay, and cost per year were identified for the 119 people on the PDSS caseload.

## RESULTS

### Question 2. Can a local alternative to OoA placements work effectively with people with PD who reach the NHS England‐defined threshold for inpatient specialist treatment?

**FIGURE 1:**
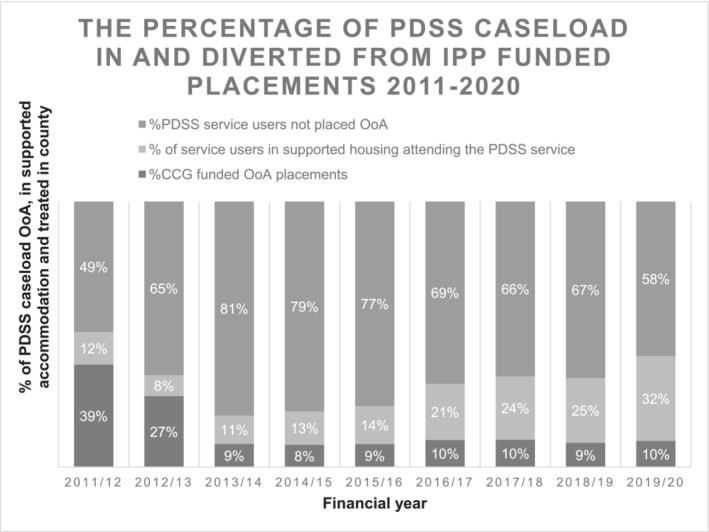
The percentage of PDSS caseload in and diverted from IPP‐funded placements.

As described, all patients on the PD service caseload reached the NHS England threshold for inpatient specialist treatment. The number of patients on the IPP and therefore PDSS caseload placed in specialist inpatient PD services OoA each year peaked at 27 in 2012/13, reducing to 12 in 2019/2020. The number of people attending the PDSS who were placed in locally supported accommodation which was partly funded by the IPP budget or remaining at home (and so not requiring IPP funding) rose from 35 in 2011/12 when the service opened to a peak of 149 in 2018/19. Figure 1 shows the number of patients using the PDSS each year who were placed out of the area or placed in locally supported accommodation or attended from home, as a percentage of all PDSS attendees to allow comparison between years.

As the number of people using the new service increased, the length of stay in OoA placements (Length of Stay [LOS]; measured in bed days) fell by 57% from a mean of 951 days (31.3 months) in 2012/13 to a mean of 406 days (13.5 months) once the PDSS was fully operational (2015–2020). This contrasts with the LOS for five people in OoA placements who, for various reasons, could not return to Devon to partake in the PDSS pathway whose LOS ranged from 33.2 months to an excess of 10 years.

#### Question 2.1. Are the costs of this alternative therapeutic pathway offset by reductions in the number of OoA placements and length of stay?

**FIGURE 2:**
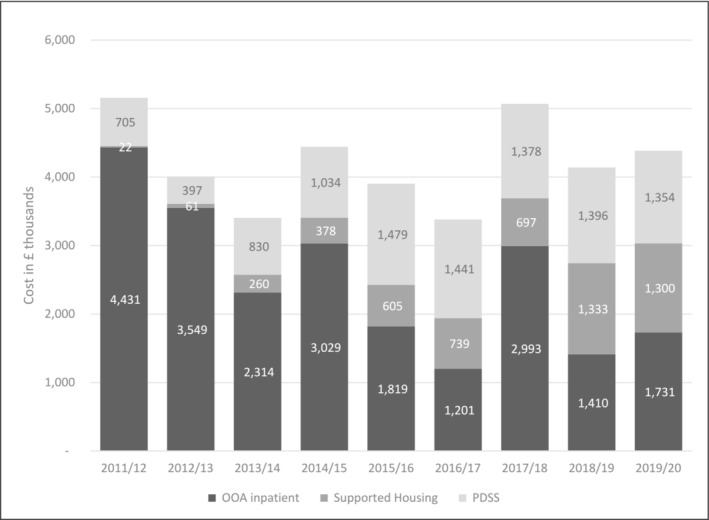
Local commissioners (CCG) spend in real terms: 2011–2020.

To examine the financial impact of introducing the new PDSS pathway, the costs to the local commissioners (CCG) of OoA placements, supported housing, and the PDSS pathway are shown in Figure 2 including the cumulative total cost.^3^ Figure 2 illustrates the phased introduction of the PDSS toward full implementation in 2014/15 and the increasing cost of supported accommodation as patients returned from OoA placement or were offered accommodation as an alternative to OoA placement. These rising local costs were offset by the reduction in OoA placement costs.

The total cost to the IPP budget and CCG including inpatient OoA placements, local supported accommodation as a step down from or alternative to OoA placement, and the cost of providing the PDSS decreased from £5,157,770 in 2011 to £4,385,561 in 2019/20, representing a 15% reduction in real terms. The reduced overall cost was spent on a larger caseload so that the average cost per case fell from a mean of £90,487 (57 cases) in 2011 to £35,947 (122 cases) in 2020, a 60% reduction. This lower cost per case arose from the lower cost of the PDSS compared to OoA placements: in 2019/2020 the average cost per OoA placement was £144,282 P/A while the average cost of PDSS placements (including supporting housing costs) was £24,129 P/A.

## DISCUSSION

The review of the national strategy and the results of the FOI request highlight the extent to which the needs of people with severe and complex PD remain hidden. In research terms, this group is an outliers, likely to be excluded from health economic and psychotherapeutic outcome studies. Their transdiagnostic presentations lead to confusion about diagnosis and difficulty identifying them for research purposes. The BIGSPD study presented here and their report highlighted this concerning lack of transparency about how many people diagnosed with PD are in OoA rehabilitation placements (Zimbron et al., 2022). Despite the expressed intentions of the Department of Health, the lack of transparency is ongoing (Department of Health & Social Care, 2016, NHS Digital, 2023).

Our findings confirm less than 20% of the total data was made available by CCGs so that we are only able to present a snapshot of current placements rather than a robust scientific analysis. Given the limitations of the data, our best estimate is that 14% of all people with Mental Health problems using OoA placements have a diagnosis of PD and the mean cost of placement for people with PD and psychosis are similar. Our findings also highlight poorly co‐ordinated pathways for people returning from OoA placement which do not appear to include local PD services. These services are in any case not resourced to offer the intensity of therapeutic input required to contain people with severe and complex PD in community settings.

A unified system for collecting data to inform a national strategy and improve services and pathways for this group of people is needed given that the most recent figures in November 2023 confirm an increase in OoA placement days from 225,190 days in October 2017 to 282,870 in November 2023 (NHS Digital, 2023). From reported data, the OoA bed days for people diagnosed with PD have actually risen from 17,670 to 17,930 for the same time period with associated costs rising by 37.8%. While this study focused on PD placements OoA these represent the tip of the iceberg in understanding the wider problem of people with PD in hospital settings. People with these difficulties are overrepresented in mental health inpatient settings (Evans et al., 2017), in acute hospital inpatient settings (Bermingham et al., 2010; Macina et al., 2021), and in specialist Eating Disorder services (Sansone et al., 2013). The absence of local psychotherapeutic services with resources to manage the risks and transdiagnostic presentations of people with severe and complex PD contributes to the over‐reliance on inpatient care.

In view of the absence of psychotherapeutic alternatives to inpatient care, we presented data from the Devon PDSS a community therapeutic service commissioned specifically to address the high use of OoA placements. Our findings confirm that a local intensive psychotherapeutic service can work effectively with people with PD who reach the NHS England defined threshold for inpatient specialist treatment reducing OoA placements and increasing the use of less restrictive local residential alternatives as well as providing a service for many patients reaching this threshold who could attend from home and therefore did not need placement at all. The cost of providing the service was offset by the reduction in the number of OoA placements and length of stay even though, as people returned within the area more supported housing placements were required. Overall costs reduced by 15% in real terms and were sustained over a nine‐year period.

Aspects of the pathway that were critical to success included setting a severity threshold and gatekeeping function to ensure the service focused on people with the most severe problems, minimizing inappropriate OoA placements and delayed discharges. A multi‐agency approach was essential and was achieved by the outreach team and medical psychotherapy consultants working alongside mental and physical health teams and housing providers to integrate care across agencies. Handoffs between services were minimized because all those reaching the threshold for the service were considered ‘our business’ and were assertively engaged. Finally, the therapeutic adaptations described within the Relational Affective Model meant that people detained under the Mental Health Act were accepted in the therapeutic program. The model also defined a formulation‐based therapeutic approach for patients with transdiagnostic presentations including self‐harm, eating disorders, functional symptoms, substance misuse, and disorders on the autistic spectrum. The results from the Devon PDSS provide proof of concept that people with severe difficulties usually managed in inpatient settings can be treated in a local outpatient psychodynamic intensive therapeutic service adapted to meet their needs.

A limitation of the PDSS study is that it only evaluated IPP budget costs and the cost of the PDSS itself. ‘In county’ costs of mental health services to people with severe and complex PD were not estimated. This is being addressed in the HEARD study (Health Economics and Relational Disorder IRAS ID. 262,622) which identified high‐cost, non‐forensic PD outliers in two NHS Trusts through a search of health and social care electronic records. The findings of this study will report all health and social care costs associated with this most severe and complex PD group providing a full picture of costs and cost savings for the PDSS caseload. Other unmeasured factors may have contributed to the reduction in OoA placements. For example, the IPP team took on care coordination for OoA patients during this period working closely with the PDSS to facilitate transition between placements. Equally unmeasured factors such as increasing pressure on community services and local inpatient beds increase the demand for OoA placements as was the case in 2017/18 (Figure 2). The functioning of the service was to an extent dependent on its multi‐agency context. The clinical outcomes of the service are not presented here.

The effectiveness of PDSS achieved these results in the context of a wider health and social care system which was not organized to address the needs of people with severe and complex PD and staff in wider services who often felt ill‐equipped to deal with their complex presentations. The effectiveness of future therapy services is likely to be augmented by provider collaboratives whose explicit purpose is to improve training, pathways, and collaborative working between agencies for people with severe and complex PD.

## CONCLUSION

The studies reported here demonstrate the high number and high costs of OoA placements for people with severe and complex PD who remain a hidden population in mental health services. The case is made that this is a neglected area in health and social care strategy, remaining largely unmonitored with high costs and poor outcomes.

Preliminary evidence for the cost effectiveness of a specialist PD pathway in Devon is presented indicating the savings made in reducing OoA placements cover the cost of the service and redistribute the resource expended on OoA placements to a wider group of people with highly complex needs. We suggest this service provides a model for a future safer and more effective therapeutic rehabilitation pathway for people with severe and complex PD.

## Footnotes

^1^
The diagnosis of Personality Disorder is mired in controversy in the UK being associated with stigmatization. The term is used in this paper because it is the current ICD‐11 terminology. To acknowledge the controversy about the term it is used in quotation marks.

^2^
OoA placements are defined as ‘appropriate’ if there are safeguarding issues, offending restrictions, the service user is employed by the local service, they become unwell when away from the local area or by service user choice (to be near family or social networks). All other placements to acute or long‐term rehabilitation placements outside the catchment of their local community mental health service where they cannot be visited regularly by their care co‐ordinator are classified as ‘inappropriate’.

^3^
The CCG costs each year have been corrected for inflation (using the Bank of England inflation calculator).
